# Boric acid and Schiff base-based fluorescent sensor for detection of L-tryptophan in milk and BSA samples

**DOI:** 10.55730/1300-0527.3381

**Published:** 2022-02-28

**Authors:** Ümran DURU KAMACI, Musa KAMACI

**Affiliations:** 1 Department of Chemistry, Faculty of Arts and Sciences, Yıldız Technical University, İstanbul, Turkey; 2 Piri Reis University, İstanbul, Turkey

**Keywords:** Tryptophan, boric acid, biosensor, fluorescent sensor, Schiff base

## Abstract

In the present paper, the fluorescence sensor based on Schiff base and boric acid was developed for easy and rapid detection of L-tryptophan in different samples such as milk and bovine serum albumin. The photoluminescence intensity was measured by using fluorescent measurements and the results indicated that the developed fluorescent sensor was exhibited selective, sensitive, reliable determination against L-tryptophan, and a series of various analytes such as cations, amino acids, and organic compounds were used to investigate the selectivity of the fluorescent chemosensor. The limit of detection and linear range of the chemosensor were calculated as 0.82 μM, and 0.1–500 μM, respectively. The performance of the chemosensor was evaluated in terms of selectivity, reversible usage, stability, and interference/anti-interference. The developed chemosensor was exhibited excellent photostability, and it was a great potential application of L-tryptophan in bovine serum albumin and milk samples.

## 1. Introduction

Boric acid (BA) is an important inorganic acid for animals, microorganisms, and plants due to its vital role [1]. It has been shown a good immune regulatory effect, antiinflammatory, antifungal, and antibacterial behavior as biomaterials, and BA has been exhibited a positive wound-healing effect [2, 3]. Also, BA has an electron-deficient group as its structural property, and it is also used for the determination of analytes due to this feature [4].

Schiff bases have been composed of azomethine or imine (−N=CH) bonding as a characteristic property [5, 6]. They have been exhibited a strong chelating ability with the ligands due to their excellent donor ability, and their complexes showed biological distinctive properties such as antibacterial, antifungal, antivirus, etc. [7, 8]. Also, they have been used as catalyst, anticorrosive, semiconductive, and thermally resistant materials [9, 10]. As a chemosensor, the imine (−N=CH) bonding in the structure of the Schiff bases has been gained the ability to easily and rapidly determine any analytes such as cations, anions, amino acids, or organic compounds, and this type of chemosensor has particularly interesting properties such as low-cost, optical performance, etc. [11, 12].

L-tryptophan (Trp) is an aromatic essential amino acid and it has an important role in protein synthesis, stability, and functions [13]. This amino acid has not been synthesized in the human body and it must be taken daily via foods [14]. In addition, excessive intake of Trp can cause some disorders such as psychiatric and sleep, and diseases such as Parkinson’s, depression, neurodegenerative, and schizophrenia [15, 16]. Recently, Trp has been detected in the blood of people with gastric cancer, but it has not been found in the blood samples of healthy people due to this property the developed sensors for the determination of the Trp level can be used to detect gastric cancer patients [15]. Moreover, another article published by Lee et al. stated that the determination of tryptophan level in biological fluids is an important signal source for early diagnosis and prevention of liver cancer patients [15]. Therefore, there is still a need for fluorescent sensors to be developed to detection of the Trp level in different samples due to these mentioned properties.

The proposed paper aimed to prepare a fluorescent chemosensor for easy and rapid detection of L-tryptophan. The proposed fluorescent chemosensor was composed of the boric acid in the Schiff bases matrix. The Schiff base was used to obtain a homogeneous matrix, and enhance the fluorescence intensity of boric acid. For this purpose, the Schiff base was prepared by the reaction of an aldehyde with a nitrogen-rich compound of amine. The fluorescence and absorbance spectra were measured in the presence of various cations (iron (II), silver (I), aluminum (III), copper (II), lithium (I), zinc (II), cobalt (II), manganese (II), sodium (I), barium (II), nickel (II), iron (III), magnesium (II), and potassium (I)), amino acids (glycine, L-cysteine, L-alanine, L-arginine, L-tryptophan, L-histidine, and L-phenyl alanine), and organic compounds (ethylenediaminetetraacetic acid, and urea). The results demonstrated that the developed chemosensor was exhibited sensitive and selective detection of Trp.

## 2. Material and methods

### 2.1. Chemical

2,4,6-triamino-1,3,5-triazine (AT), 3,5-di-tert-butyl-4-hydroxybenzaldehyde hemihydrate (BHBA), ethanol (EtOH), acetonitrile (MeCN), iron (II) chloride (Fe^2+^), silver nitrate (Ag^+^), aluminum chloride (Al^3+^), copper (II) chloride (Cu^2+^), lithium sulfate (Li^+^), zinc chloride (Zn^2+^), cobalt (II) chloride (Co^2+^), manganese sulfate (Mn^2+^), sodium chloride (Na^+^), barium chloride (Ba^2+^), nickel (II) chloride (Ni^2+^), iron (III) chloride (Fe^3+^), magnesium sulfate (Mg^2+^), potassium chloride (K^+^), glycine (Gly), L-cysteine (Cys), L-alanine (Ala), L-arginine (Arg), L-tryptophan (Tyr), L-histidine (His), and L-phenyl alanine (Phe), ethylenediaminetetraacetic acid (EDTA), and urea were commercially supplied and they were used without any purification.

### 2.2. Synthesis of TAMP

**Figure 1:**
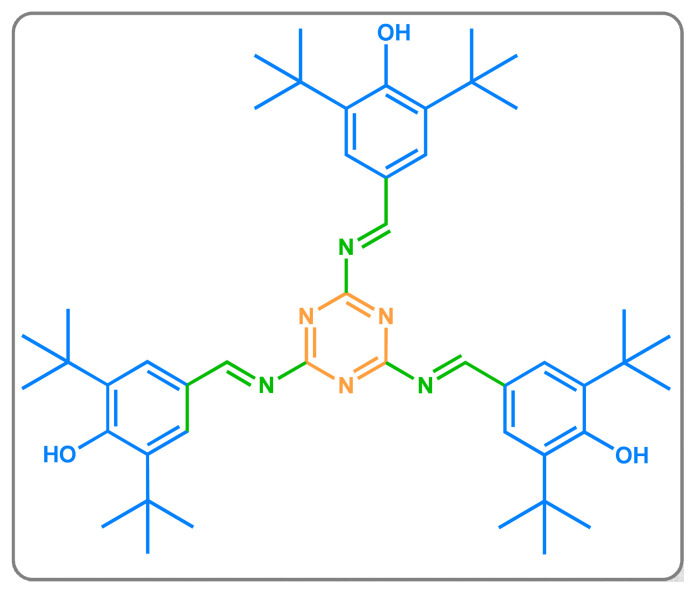
Structure of TAMP.

Schiff base was abbreviated as TAMP (Figure 1) and it was prepared by the reaction of BHBA with a nitrogen-rich compound (AT) in EtOH/distilled water mixture (4:5; v:v) at 70 °C in an oil bath for 4 h. After the reaction mixture was placed into the Petri dish and solvent of the mixture was evaporated. Then, TAMP was washed with distilled water (2 × 50 mL) and MeCN (2 × 25 mL), and it was dried in a vacuum oven around 60 °C overnight [17]. The yield of the Schiff base was calculated as follows [18]:


$$\begin{array}{l} \text{Yield }(\%)=\frac{The\;obtained\;Schiff\;base\;amount}{The\;starting\;amount}\times 100.\\ \text{Yield:}\;98\%,\;\text{appearance:}\;\text{white powder}. \end{array}$$

### 2.3. Preparation of the stock solution

A total of 50 mM stock solutions of TAMP and boric acid were prepared in 250 mL ethanol, and 100 mL distilled water by using an ultrasonic bath, respectively. A total of 50 mM stock solutions of the tested metal cations (Fe^2+^, Ag^+^, Al^3+^, Cu^2+^, Li^+^, Zn^2+^, Co^2+^, Mn^2+^, Na^+^, Ba^2+^, Ni^2+^, Fe^3+^, Mg^2+^, and K^+^), amino acids (Gly, Cys, Ala, Arg, Tyr, His, and Phe), and organic compounds (EDTA, and urea) were prepared in distilled water.

### 2.4. Instruments

The characterization of TAMP was carried out by using FT-IR (Perkin-Elmer Spectrum Two FT-IR Spectrometer), ^1^H, and ^13^C-NMR (Agilent 600 MHz Premium COMPACT NMR Magnet), and the results were given in Supplementary Materials. Absorption spectra of boric acid (BA), TAMP, the mixture of TAMP and BA in the presence and absence of tryptophan (Trp) were recorded by using a Perkin-Elmer UV-Vis spectrophotometer in the range from 250 to 700 nm. Fluorescence measurements were carried out by using a Perkin-Elmer LS 55 fluorescence spectrophotometer between 350 and 800 nm. Quartz cuvette with 10×10 mm light path was used in fluorescent measurements, and excitation wavelength and slid with were adjusted as 390 and 5 nm in these measurements, respectively. ^1^H-NMR spectra of the TAMP-BA mixture in the presence and absence of Trp were recorded using a Bruker AC400 FT-NMR spectrometer. Also, FT-IR spectra of TAMP, BA, Trp, the mixture of TAMP-BA in the presence and absence of Trp were measured on a Nicolet iS10 Thermo Scientific FT-IR spectrometer with an ATR device.

### 2.5. UV-Vis and fluorescence analysis procedure

Fluorescence and UV-Vis measurements were performed as follows: 900 μL of TAMP (50 mM) and 50 μL of BA (50 mM) were mixed in a test tube and the mixture was incubated for 5 min under the ambient conditions. Then, 50 μL of analyte (metal ions, amino acids, and organic compounds) was added into the test tube and the prepared mixture was incubated for 10 min.

## 3. Result and discussion

### 3.1. Photophysical properties

**Figure 2:**
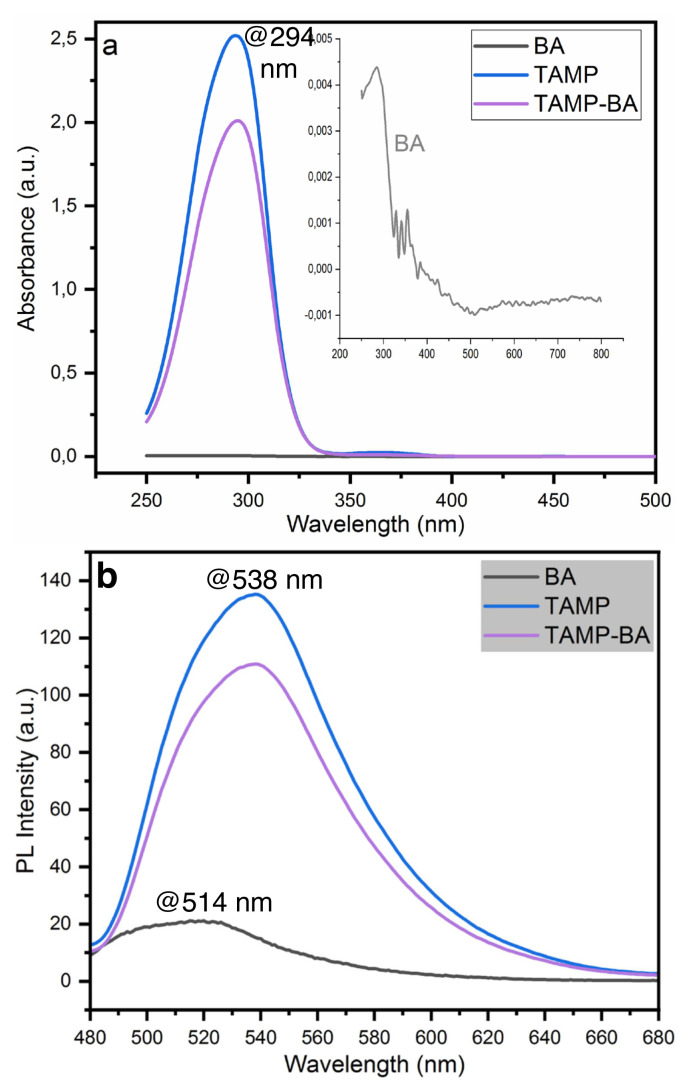
UV-Vis (a), PL (b) spectra of TAMP, BA, and TAMP-BA.

Photophysical properties of the fluorescence probe were investigated by using UV-Vis and fluorescence spectrophotometers (Figure 2). As can be seen in Figure 2a, TAMP was showed a strong absorbance peak around 294 nm due to π→π* electronic transition of imine bonding in the structure of this compound [19, 20], and boric acid (BA) was exhibited a low absorption behavior. Upon addition of BA into TAMP solution, the absorption spectrum of TAMP-BA was decreased around 0.80-fold.

According to PL spectra TAMP and BA, they were showed fluorescence wavelength around 538 and 514 nm, respectively (Figure 2b). Upon addition of BA into TAMP solution fluorescence intensity was decreased 0.79-fold.

### 3.2. Selectivity and detection of L-tryptophan

**Figure 3:**
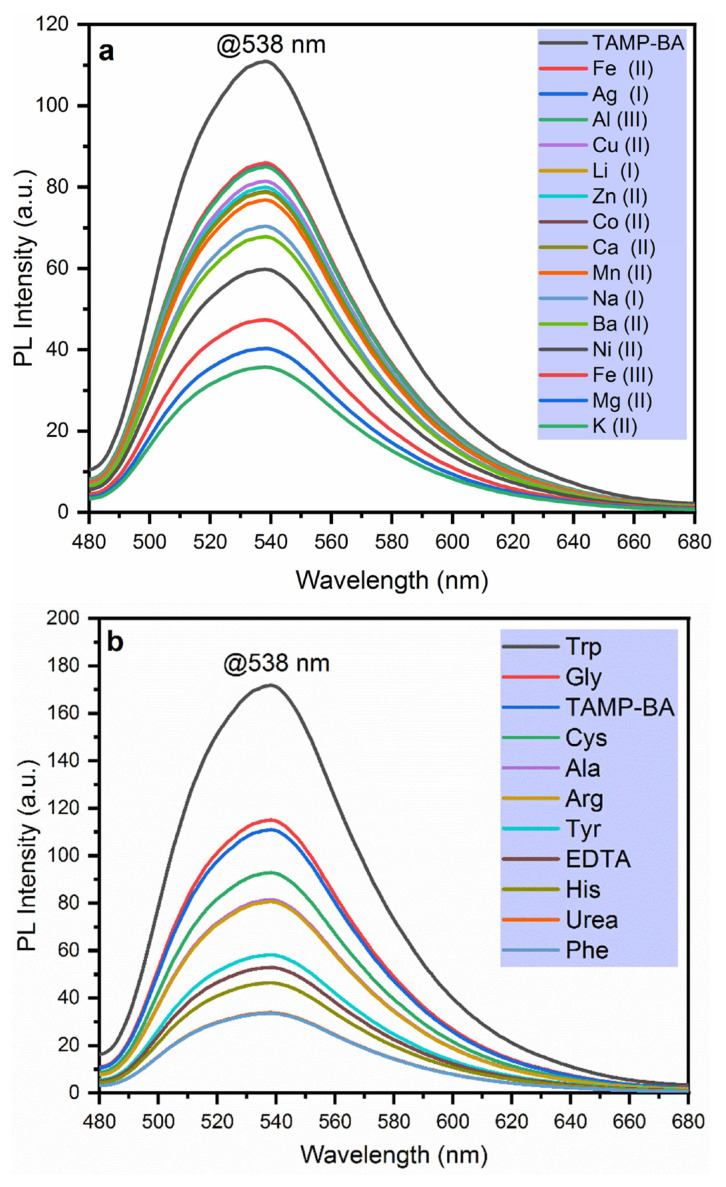
Emission intensity change of TAMP-BA fluorescent probe in the presence of 50 mM of different metal ions (a), amino acids, and organic compounds (b).

Figure 3 represents fluorescence spectra of TAMP-BA in the presence of the analytes such as metal cations, amino acids, and organic compounds. The fluorescence change of the proposed chemosensor in the presence of the various metal cations such as Fe^2+^, Ag^+^, Al^3+^, Cu^2+^, Li^+^, Zn^2+^, Co^2+^, Mn^2+^, Na^+^, Ba^2+^, Ni^2+^, Fe^3+^, Mg^2+^, and K^+^ was demonstrated in Figure 3a. As can be seen, the fluorescence intensity of the chemosensor was decreased in the range of 0.77 fold (Fe^2+^, Ag^+^, and Al^3+^) to 0.32-fold (K^+^) with the addition of cations solution into the TAMP-BA mixture at 538 nm. Moreover, Figure 3b demonstrates fluorescence intensity change of the developed chemosensor in the presence of amino acids such as amino acids (Gly, Cys, Ala, Arg, Tyr, His, and Phe), and organic compounds (EDTA, and urea). Fluorescence intensity value was recorded as 171.81, 114.99, 92.81, 81.44, 80.63, 58.17, 52.90, 46.40, 33.82, and 33.55 a.u. for Trp, Gly, Cys, Ala, Arg, Tyr, EDTA, His, urea, and Phe at 538 nm, respectively.

According to these results, Trp (1.55-fold) and Gly (1.04-fold) have higher emission intensity than TAMP-BA while the other tested amino acids and organic compounds have lower emission intensity. The results indicated that TAMP-BA fluorescent probe was exhibited a good selectivity response toward Trp.

### 3.3. The fluorescence sensing of Trp

**Figure 4:**
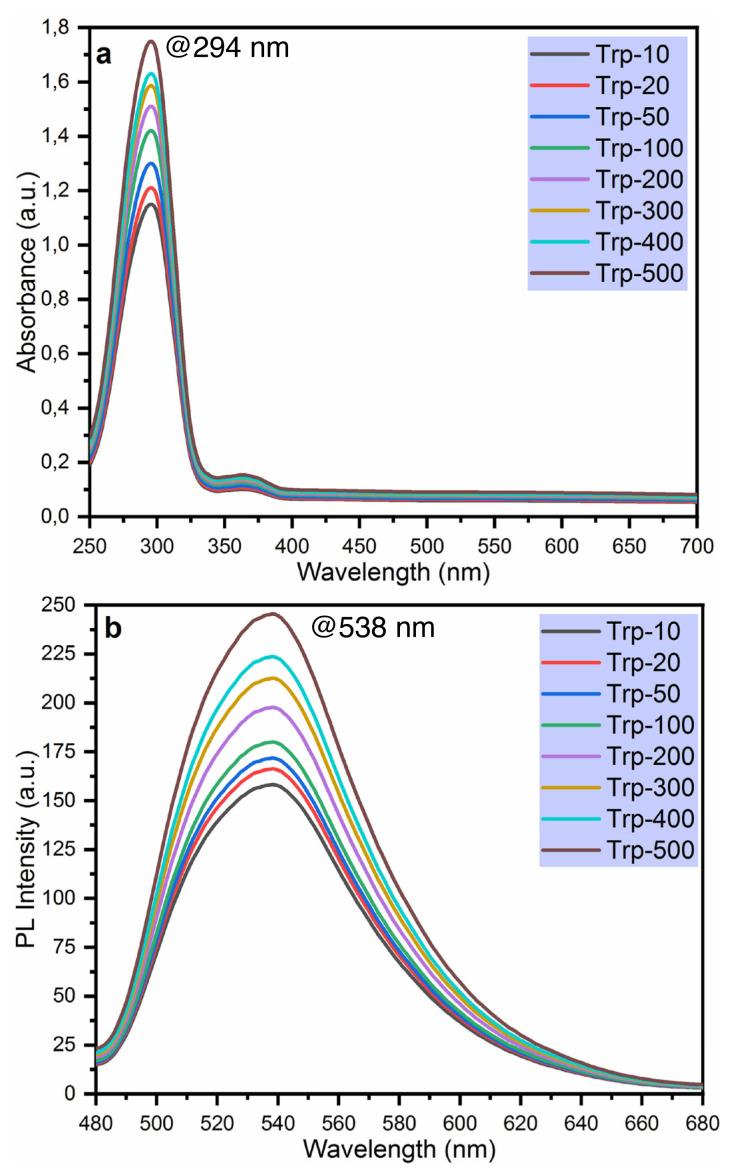
Change in absorbance (a) and photoluminescence (b) intensity in the presence of 50 μM TAMP and different concentrations of Trp.

To examine its spectral efficiency and potential applicability of the developed sensor for the detection of L-tryptophan, the absorbance and fluorescent spectra of TAMP-BA were measured in the presence of various Trp concentrations, and they were given in Figures 4a and b, respectively. These measurements were performed in the presence of 900 μL 50 μM TAMP, 50 μL 50 μM boric acid, and 50 μL L-tryptophan at various concentrations in the range from 10 to 500 μM. As can be seen in Figures 4a and 4b, absorbance and emission intensity were gradually increased in the presence of the used Trp concentration from 1.15 (10 μM) to 1.75 (500 μM) a.u., and 158.24 (10 μM) to 245.52 (500 μM) a.u. at 538 nm, respectively. These results exhibited that the TAMP-BA fluorescent sensor can be successfully applied for the determination of L-tryptophan.

**Figure 5:**
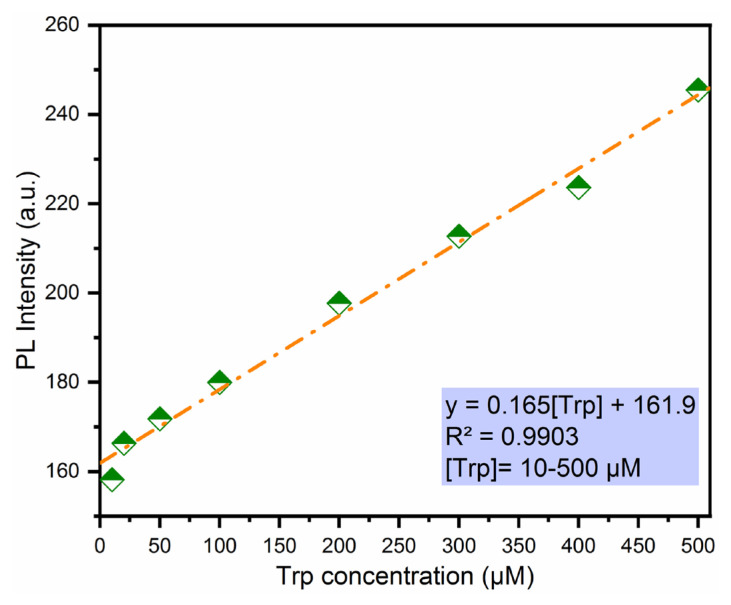
The linearity between the different concentrations of Trp and PL intensity at 538 nm.

Moreover, a calibration curve between the different Trp concentrations in the range from 10 to 500 μM and the fluorescent chemosensor was obtained to clarify the practical applicability by using the titration experiment (Figure 5). The experiment results revealed that the fluorescent intensity of the developed chemosensor was gradually increased with the increasing L-tryptophan concentrations in the mentioned concentration ranges with good linearity (R^2^ = 0.9903).

### 3.4. Limit of detection and stoichiometry

The limit of detection (LOD) of the fluorescence chemosensor was calculated by using the fluorescence titration method as the following equation [21, 22]:


$$\text{LOD}=3\sigma_{bi}/\text{m},$$

where σ_bi_ and m are the standard deviation of the fluorescent chemosensor based on TAMP and boric acid solution and slope (Figure 5), respectively. The regression equation was found as F = 0.165[Trp] + 161.9. LOD value of the developed sensor was calculated as 0.82 μM.

**Figure 6:**
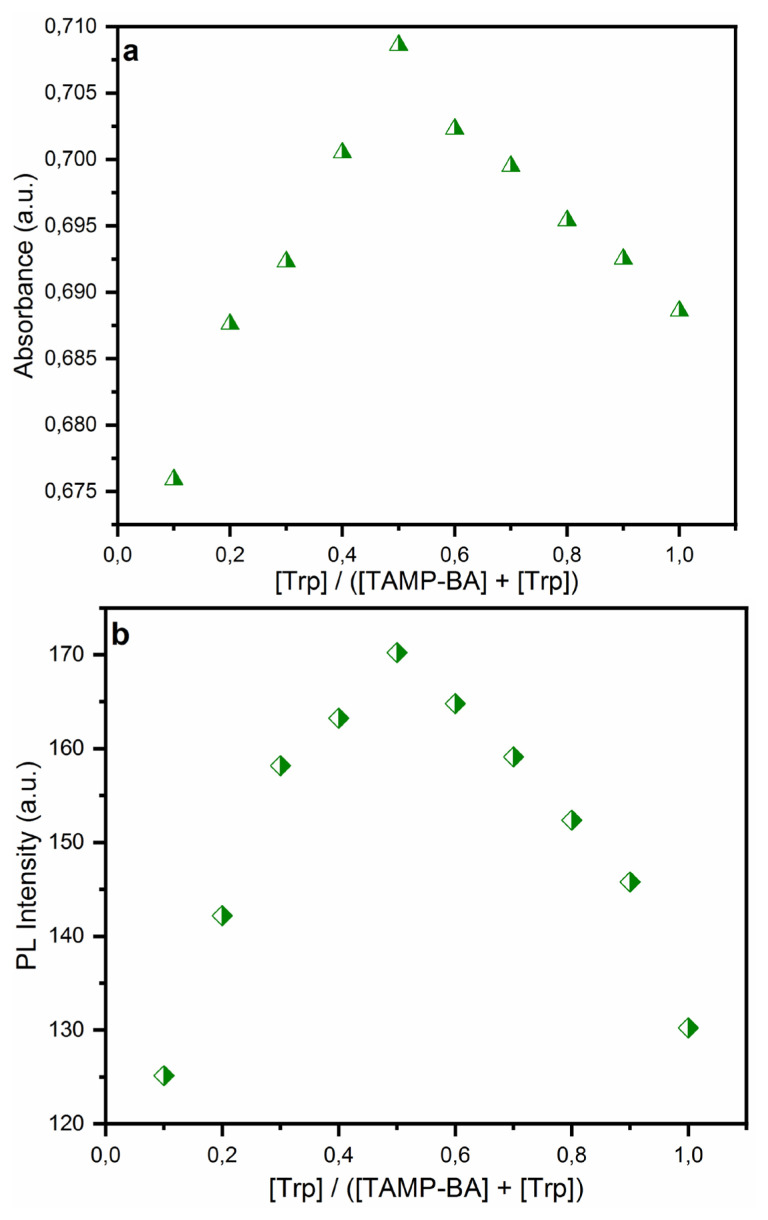
UV-Vis (a), and PL (b) Job’s plot for complexation of TAMP-BA with Trp in distilled water at 538 nm.

The stoichiometry between TAMP and BA-based chemosensor and L-tryptophan was studied using the Job’s plot method both UV-Vis and PL spectroscopy (Figure 6). As can be seen in Figure 6, the developed chemosensor was exhibited maximum absorbance and emission intensity at $\frac{\left[Trp\right]}{\left[Trp]+[Probe\right]}=0.5$. These results indicated that the binding ratio between the probe and Trp was determined as 1:1.

The TAMP and boric acid-based chemosensor was compared to the reported paper in literature for the detection of Trp with various methods such as electrochemical and fluorescence (Table 1). The results showed that the selectivity and LOD value of the developed chemosensor in this paper was comparable with the other studies or they were better than some reported papers [23–29].

### 3.5. Characterization of TAMP-BA chemosensor in the presence of Trp

**Figure 7:**
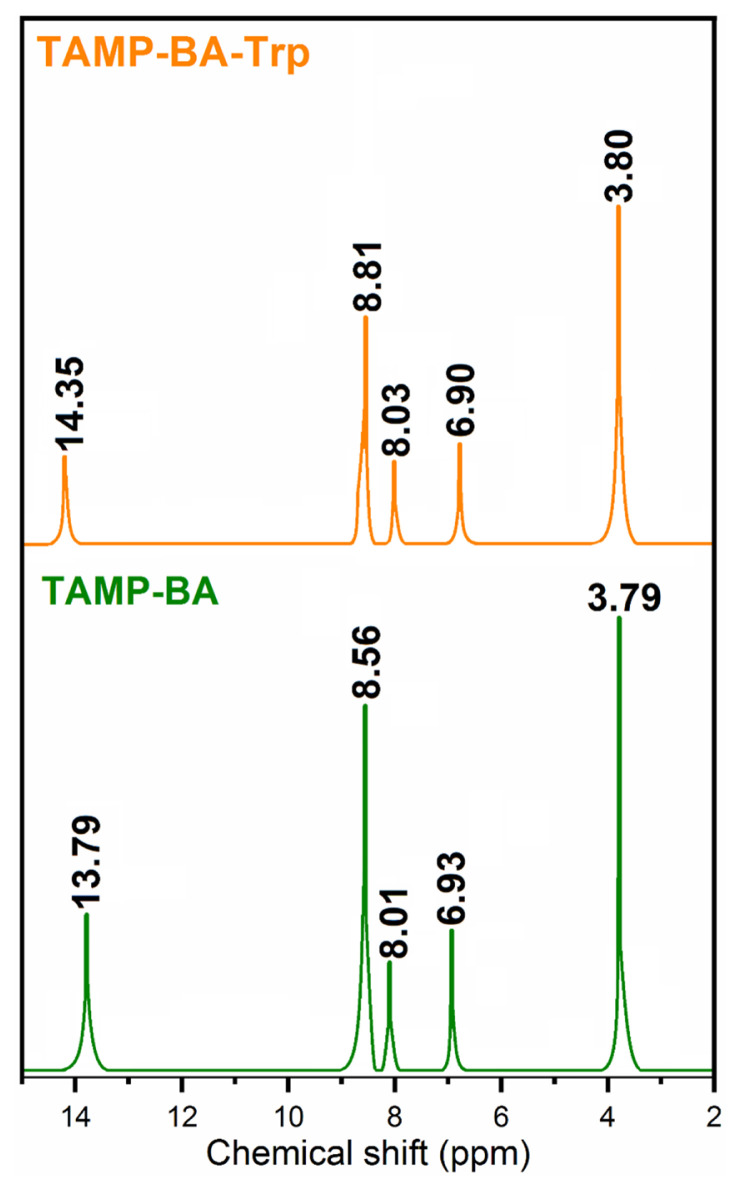
^1^H-NMR titration of TAMP-BA in the presence and absence of Trp in DMSO-*d_6_*.

**Figure 8:**
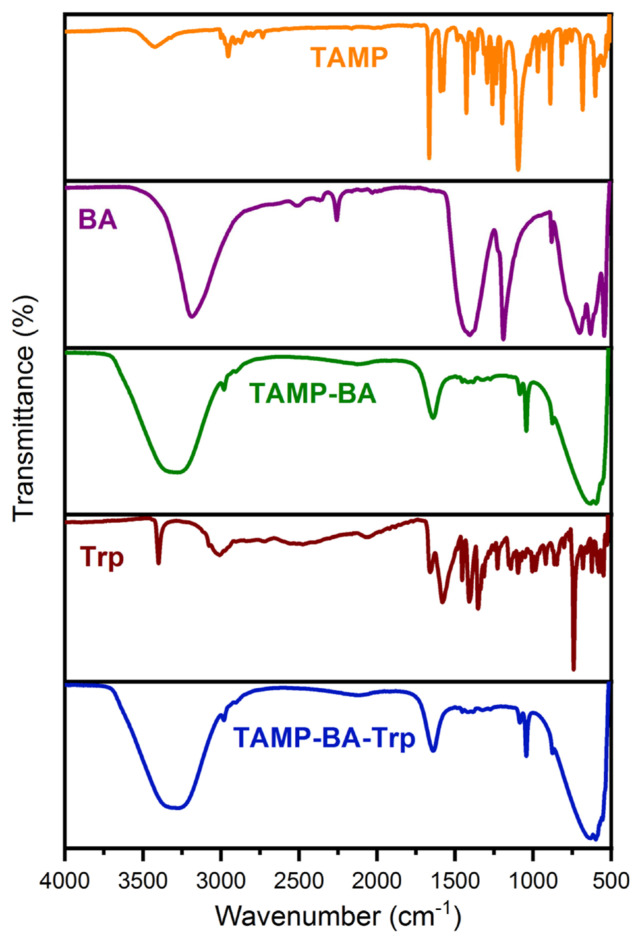
FT-IR spectra of TAMP, boric acid (BA), TAMP-BA, tryptophan (Trp), and TAMP-BA-Trp.

The characterization of the fluorescent sensor (TAMP-BA) in the presence of the Trp was performed by using NMR (Figure 7), and FT-IR (Figure 8) analysis techniques.

The complex interaction between the TAMP and boric acid-based chemosensor and Trp was also performed using the ^1^H-NMR titration method in DMSO-*d_6_* (Figure 7). Hydroxyl (C-OH) and imine (−N=CH) protons of TAMP-BA were observed at 13.79, and 8.56 ppm, respectively. These proton peaks were shifted to upfield (14.35, and 8.81 ppm) after the addition of Trp into the TAMP-BA solution due to the strong hydrogen bonding interaction of amine nitrogen (−NH_2_) or carboxylic acid oxygen (−COOH) of Trp with azomethine nitrogen (−N = CH) or hydroxyl oxygen (−OH) of TAMP [30].

To investigate the interaction between the developed chemosensor and tryptophan FT-IR spectra of the compounds and mixture were measured (Figure 8). In the FT-IR spectrum of TAMP, the characteristic hydroxyl (−OH), imine (−N = CH), and aliphatic −CH (Al-CH) stretching vibrations were recorded at 3423, 1593, and 2956 cm^−1^, respectively [17]. The hydroxyl (−OH), asymmetric −B-O, B-O-H, and symmetric B-O stretching vibrations were detected at 3187, 1416, 1189, and 717 cm^−1^, respectively [31]. These stretching vibrations in the structure of TAMP-BA were seen at 3306 (−OH), 1651 (−N=CH), 1405 (asymmetric B-O), 1077 (B-OH), and 634 (symmetric B-O) cm^−1^, respectively. As can be seen FT-IR spectrum of the pristine tryptophan, −N-H, carbonyl (−C=O), and Al-CH stretching vibrations were recorded at 3405, 1658, and 3021 cm^−1^, respectively [32]. Also, −OH, Al-CH, −N=CH, asymmetric B-O, B-OH, and symmetric B-O stretching vibrations were observed at 3305, 2987, 1634, 1414 1045, and 645 cm^−1^, respectively, whereas the other peaks such as −C=O and −N-H stretching of Trp, and B-OH in the structure of TAMP-BA-Trp were not seen due to overlapping of these bands between imine and hydroxyl stretching. These results showed that stretching vibrations of TMAP-BA were shifted to lower values after the addition of Trp due to the formation of hydrogen bonding between the fluorescent chemosensor and Trp [33].

### 3.6. Interference

**Figure 9:**
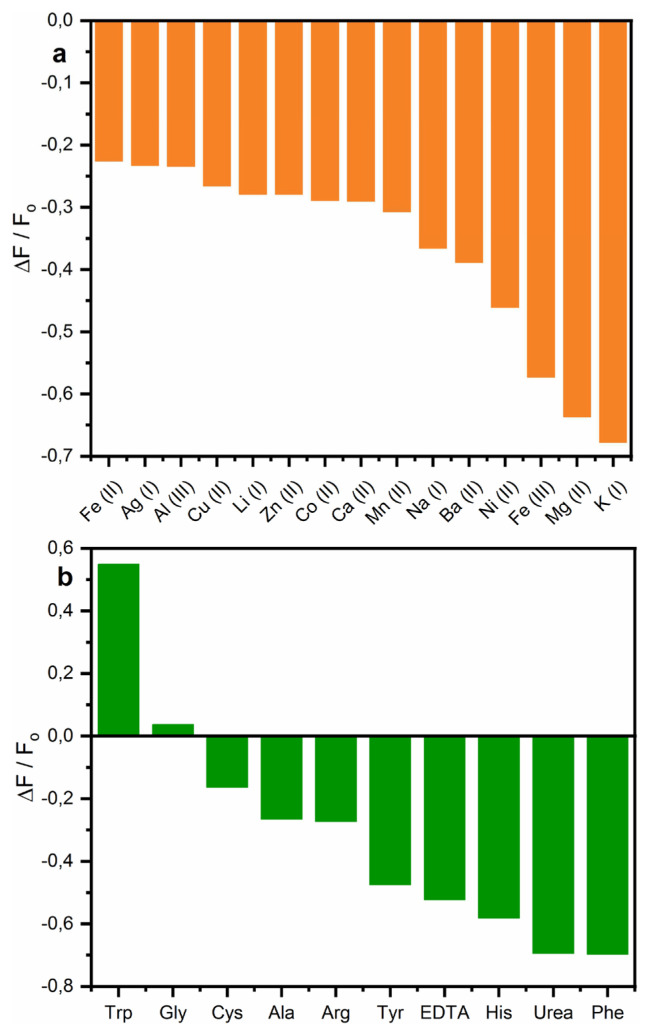
The interference effect of the used cations (a), amino acids, and organic compounds (b) on the TAMP-BA sensor in the presence of Trp at 538 nm.

The parameters used to evaluate the performance of a sensor are selectivity, reversible usage, stability, and interference effect of the analytes. Figure 9 represents the interference or anti-interference effect of the used analytes such as cations (Fe^2+^, Ag^+^, Al^3+^, Cu^2+^, Li^+^, Zn^2+^, Co^2+^, Mn^2+^, Na^+^, Ba^2+^, Ni^2+^, Fe^3+^, Mg^2+^, and K^+^), amino acids (Gly, Cys, Ala, Arg, Tyr, His, and Phe), and organic compounds (EDTA, and urea) at 538 nm. According to Figure 9a, all of the tested metal cations have lower relative intensity than the fluorescent chemosensor, and the interference effect on the developed sensor for the detection of Trp is negligible. As can be seen in Figure 9b, Trp has around 15-fold relative intensity than Gly, and the other tested amino acid and organic compounds have negative relative intensity values. These results demonstrated that the tested analytes did not show a significant fluorescence change.

### 3.7. Reversible usage

**Figure 10:**
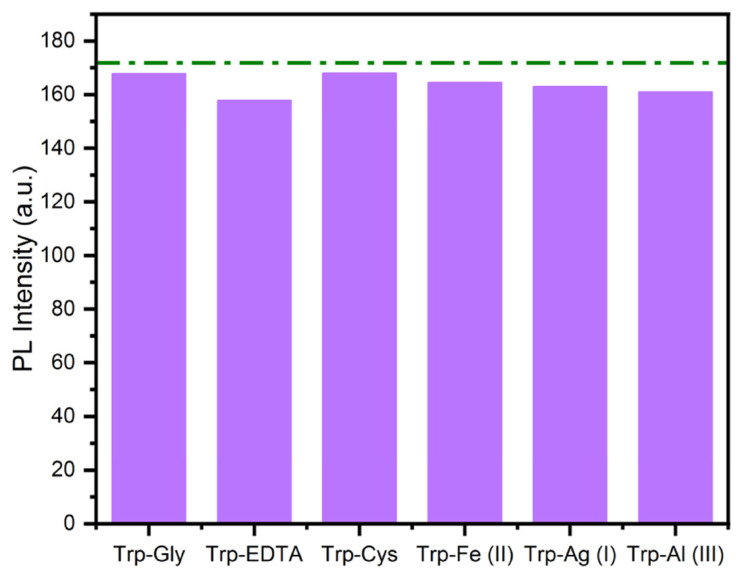
Reversible fluorescence response of TAMP-BA to Trp in the presence of Gly, EDTA, Cys, Fe (II), Ag (I), and Al (III) at 538 nm.

One another important and prerequisite parameter for a fluorescent chemosensor is reversibility due to its practical applications. For this purpose, the reversibility studies of the developed chemosensor were carried out in the presence of 50 μL of the tested analytes such as Gly, EDTA, Cys, Fe (II), Ag (I), and Al (III) (Figure 10). These six compounds were chosen due to they were the closest to the emission intensity of the chemosensor. The emission intensity of TAMP and BA-based sensor in the Trp (171.81 a.u.) was detected as 167.81, 157.82, 168.10, 164.52, 162.99, and 160.93 a.u. for Gly, EDTA, Cys, Fe (II), Ag (I), and Al (III) at 538 nm, respectively. These results indicated the chemosensor was almost completely recovered in the presence of the tested analytes due to the emission intensity being varied very little, and it could be successfully applied the detection of Trp because of this excellent property.

### 3.8. Photostability

**Figure 11:**
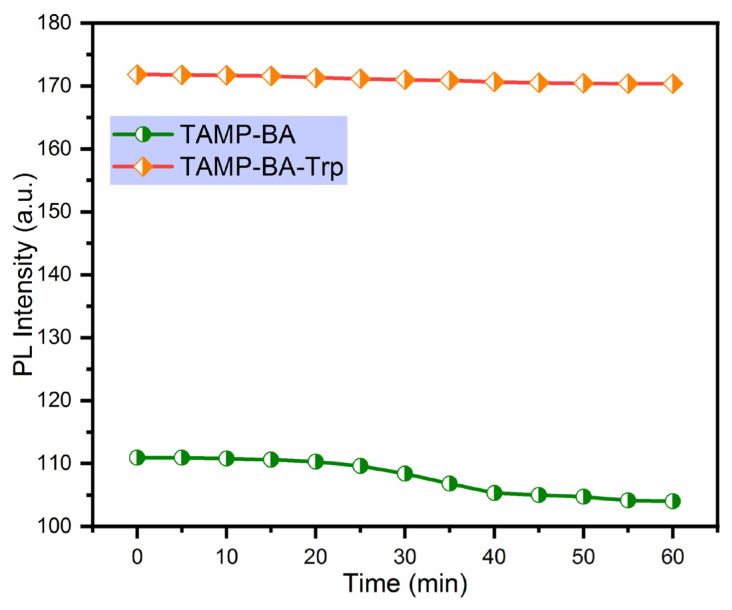
Photostability of the fluorescence chemosensor in the presence and absence of Trp at 538 nm.

Another desirable parameter is stability to evaluate the performance of the fluorescent sensor. The photostability of the TAMP-BA-based chemosensor was determined for 60 min by using 900 μL TAMP, 50 μL BA in the presence and absence of 50 μL Trp in distilled water, and the obtained curves were summarized in Figure 11 at 538 nm. As shown in Figure 11, TAMP-BA was lost around 6% of its emission intensity while TAMP-BA-Trp was lost around 1% of its emission intensity after 60 min. These results showed that the developed sensor for the detection of Trp was exhibited excellent photostability.

### 3.9. Real sample applications

To investigate the practical applications of the developed chemosensor, the TAMP-BA-based sensor was utilized to the determination of Trp in bovine serum albumin (BSA) and milk with/without lactose samples at ambient conditions in distilled water under the same measurement conditions (900 μL TAMP, 50 μL BA, and 50 μL Trp). The milk and BSA sample was added to the fluorescent chemosensor (TAMP-BA) solution and the emission intensity of the mixture was measured (Table 2). As can be seen in Table 2, recoveries were found in the range 99.25% to 100.25%, 99.50% to 101.00%, and 100% to 101.25% for BSA, milk with lactose, and milk without lactose samples, respectively. Besides, the relative standard deviation (RSD) values were also determined in the range from 2.45% to 3.20%, 2.58% to 3.73%, and 2.81% to 3.92% for BSA, milk with lactose, and milk without lactose samples, respectively. These results indicated that the developed chemosensor for the detection of Trp in actual milk and BSA samples could be successfully applied with reliable, feasibility, practical and satisfying results.

## 4. Conclusion

The fabrication of the fluorescent sensor for easy, rapid, and reliable detection of amino acids is more and more important due to the critical roles of these compounds. Herein, Schiff bases and boric acid-based on fluorescent chemosensor was developed, and it was used to determination of Trp. The limit of detection of the chemosensor was calculated as 0.82 μM, and the linear range was recorded between 0.1 and 500 μM. The stoichiometry between the fluorescent chemosensor and Trp was investigated using the Job’s plot method and the binding ratio was found as 1:1. Also, the interaction between the chemosensor and Trp was analyzed by using ^1^H-NMR titration. The results revealed that the interaction was carried out via the strong hydrogen bonding interaction of amine nitrogen (−NH_2_) or carboxylic acid oxygen (−COOH) of Trp with azomethine nitrogen (−N=CH) or hydroxyl oxygen (−OH) of Schiff base. The performance of the chemosensor was evaluated in terms of selectivity, reversible usage, stability, and interference/antiinterference. The interference effect of the tested cations, amino acids, and organic compounds on the developed sensor was negligible. The developed sensor was also showed excellent photostability for 60 min. The analytical application of the chemosensor was investigated in BSA and milk samples and the results showed that it was a great potential for the detection of Trp in these samples.

## Figures and Tables

**Table 1:** Performance comparison of TAMP-BA-based fluorescent chemosensor with the other studies.

Material	LOD	Method	References
Hydroxyapatite/graphene oxide	5.50 μM	Electrochemical	23
Ta_2_O_5_/rGO Nanocomposite	0.84 μM	Electrochemical	24
Hydrothermal carbon spheres	5.00 μM	Fluorescence	25
1,8-pyrenedione	0.15 μM	Fluorescence	26
Fe_3_O_4_/carbon composite	0.26 μM	Fluorescence	27
Carbazole	0.31 μM	Fluorescence	28
BINOL monoaldehydes	5.68 μM	Fluorescence	29
TAMP and boric acid	0.82 μM	Fluorescence	This study

**Table 2:** The application of the developed sensor (50 mM) to the detection of Trp (20 μM) in BSA and milk samples (n = 3).

Sample	Foreign substance (μL)	Recovery (%)	RSD (%)
1	BSA (50)	99.25	2.45
2	BSA (250)	100.25	3.20
3	BSA (500)	99.75	2.76
4	Milk (50)	100.50	3.48
5	Milk (250)	99.50	2.58
6	Milk (500)	101.00	3.73
7	Lactose-free milk (50)	100.00	2.81
8	Lactose-free milk (250)	100.75	3.69
9	Lactose-free milk (500)	101.25	3.92
